# Noncanonical functions of microRNAs in the nucleus

**DOI:** 10.3724/abbs.2023268

**Published:** 2024-01-03

**Authors:** Jiayi Gu, Yuanan Li, Youtong Tian, Yehao Zhang, Yongjun Cheng, Yuanjia Tang

**Affiliations:** 1 College of Basic Medical Sciences Shanghai Jiao Tong University School of Medicine Shanghai 200001 China; 2 Department of Rheumatology the First People’s Hospital of Wenling Wenling 317500 China; 3 Shanghai Institute of Rheumatology/Department of Rheumatology Renji Hospital Shanghai Jiao Tong University School of Medicine Shanghai 200001 China; 4 State Key Laboratory of Oncogenes and Related Genes Shanghai Cancer Institute Renji Hospital Shanghai 200031 China

**Keywords:** microRNA, nuclear localization, miRNA-inducing silencing complex (miRISC), gene regulation, transcriptional control

## Abstract

MicroRNAs (miRNAs) are small noncoding RNAs (ncRNAs) that play their roles in the regulation of physiological and pathological processes. Originally, it was assumed that miRNAs only modulate gene expression posttranscriptionally in the cytoplasm by inducing target mRNA degradation. However, with further research, evidence shows that mature miRNAs also exist in the cell nucleus, where they can impact gene transcription and ncRNA maturation in several ways. This review provides an overview of novel models of nuclear miRNA functions. Some of the models remain to be verified by experimental evidence, and more details of the miRNA regulation network remain to be discovered in the future.

## Introduction

MicroRNAs are a category of endogenous, noncoding small RNAs that have approximately 19–24 nucleotides in length and regulate gene expression through various mechanisms. Since their discovery in 1993 [1], extensive research has been conducted. miRNAs play a critical role in many metabolic processes, including cell differentiation, lineage specification, reprogramming, immune response, and the cell cycle [ 2‒ 6]. Moreover, miRNAs are well known to be closely linked to various kinds of diseases, including cancer [ 7‒ 9]. Given their association with numerous pathological processes, miRNAs possess significant potential as biological drug targets, and tissue-specific miRNAs can also serve as biomarkers [ 10, 11]. For medical research in this field, it is worth noting that numerous miRNAs are evolutionarily conserved [12], permitting researchers to investigate them using Drosophila, *Mus musculus*, and even plant models.

Originally, miRNAs were believed to regulate gene expression in a negative manner posttranscriptionally in the cytoplasm. In this conventional pathway, the transcription of a primary miRNA (pri-miRNA) occurs from a miRNA gene, aided by RNA polymerase II (Pol II) or Pol III [ 13, 14]. Next, Drosha and DiGeorge syndrome critical region 8 (DGCR8) cleave the pri-miRNA into pre-miRNA, which is then exported to the cytoplasm with the help of Exportin-5 [ 15‒ 18]. In the cytoplasm, Dicer cleaves the pre-miRNA into a miRNA duplex [ 13, 14], which is unwound by cytoplasmic Argonaute (Ago) protein. One strand is loaded into the miRISC, while the other strand is degraded [19]. Subsequently, miRISC serves as a negative regulator to mediate translational repression or mRNA degradation [ 13, 14].

The recruitment of miRNAs to their targets depends mainly on the sequence complementarity between them. The canonical miRNA-target interactions are mediated by the seed sequence, which is a region of 6–8 nucleotides on the 5′ end of the miRNA that forms Watson-Crick base pairs with the target [20]. Noncanonical miRNA-target interactions also exist in the functions of numerous miRNAs, such as miR-24 and let-7 [ 21, 22]. Some of these interactions do not follow simple seed sequence pairing and contain multiple mismatches, bulges and wobbles, indicating that miRNA targeting modes may be complex and flexible.

**Figure 1:**
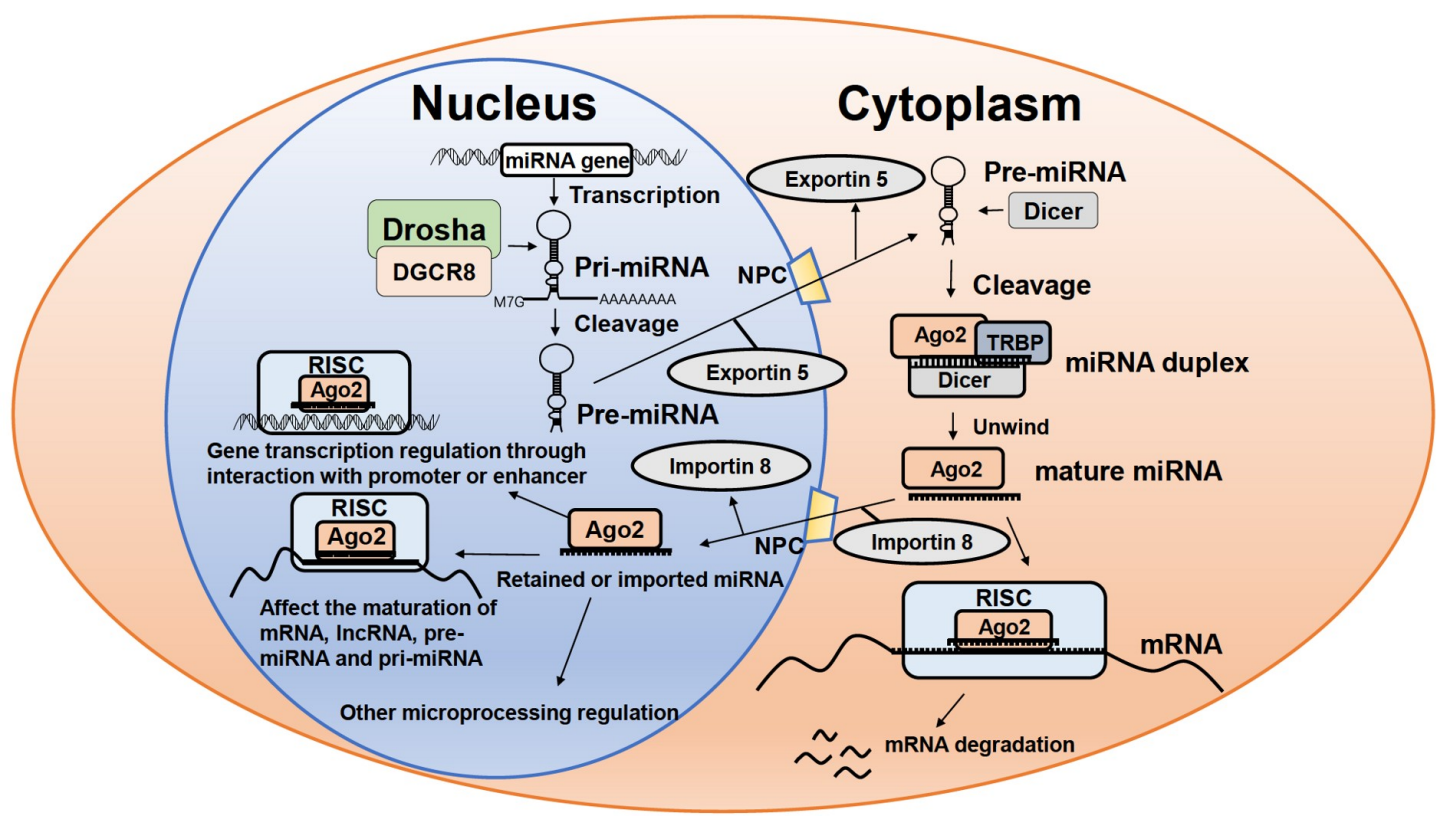
Figure 1 Biogenesis and functions of miRNAs Primary miRNAs (pri-miRNAs) are transcribed from miRNA genes by Pol II or Pol III. They are cleaved by Drosha and DGCR8 into precursor miRNAs (pre-miRNAs), which are then exported into the cytoplasm with the help of Exportin-5. Pre-miRNAs are cleaved into miRNA duplexes by Dicer and unwound by cytoplasmic Argonaute protein. One strand of the miRNA duplex is loaded into miRISC to repress mRNA expression by inducing degradation of mRNA. A proportion of mature miRNAs are translocated to the nucleus with the help of Importin-8 (IPO8). Their newly recognized functions include regulating gene transcription through interaction with promoters or enhancers, regulating the maturation of mRNA, lncRNA, pre-miRNA and pri-miRNA and other microprocessing engagement.

Over time, evidence has emerged supporting the existence of mature miRNAs in the nucleus [ 23 ‒ 28]. Numerous mechanisms of nuclear miRNA functions have been discovered, including their interactions with DNA, RNA, and proteins [ 29‒ 32], which suggests that nuclear miRNAs play a critical role in the overall miRNA-related gene regulation network ( Figure 1). This review primarily concentrates on nuclear functional miRNAs, summarizing research conducted in this field in recent years.

## Presence of miRNA in the Nucleus

An accumulating body of evidence indicates the presence of mature miRNAs in the cell nucleus. The first identified nuclear miRNA was miR-21 [23], which has since prompted numerous studies exploring the localization and functions of miRNAs in the nucleus [ 33‒ 35]. With the application of high-throughput profiling technologies, researchers have been able to compare and contrast the distribution of miRNAs between the nucleus and cytoplasm. The results of these studies indicate that most miRNAs that are present in the cytoplasm can also be found in the nucleus, with varying levels of abundance [27].

It is speculated that cells may transport not only miRNAs but also miRNA effector molecules, such as Ago proteins, to the nucleus to enhance the efficiency of miRNA-mediated gene regulation [36]. In the conventional pathway of miRNA-mediated gene regulation, miRNAs and Ago proteins operate together in the cytoplasm [19]. Consistent with the presence of miRNAs in the nucleus, numerous studies have also reported the existence of Ago proteins in this compartment [37]. Moreover, it has been demonstrated that Ago proteins play a role in nuclear miRNA function through the formation of miRISCs [37].

Although the exact mechanism of miRNA nuclear translocation remains unknown, research shows that nuclear miRISC is 20-fold smaller than its cellular counterpart [38]. When imported into the nucleus, some cofactors, such as Dicer, are not attached to the miRISC, while core components, Ago2 and Trinucleotide Repeat Containing Adaptor 6 (TNRC6), are conserved both in the nucleus and cytoplasm [ 38, 39]. Since these components exist both in nuclear miRISCs and cellular miRISCs, it is supposed that they are functional in miRNA translocation.

A previous study showed that TNRC6A plays an important role in the nuclear translocation of Ago2 and miRNA. It has been reported that TNRC6A serves as a nuclear-cytoplasmic shuttling protein that can bring Ago2 into the nucleus with its Ago-interacting motifs [40].

It has also been suggested that Importin-8, a member of the karyopherin family, is critical for mediating the cytoplasm-to-nuclear transport of mature miRNAs [41]. Experiments have shown that knocking down *IPO8* reduces the level of miRNAs and Ago2 inside the nucleus without affecting their total cellular level, indicating that their nuclear translocation is suppressed [42]. Additionally, through cross-immunoprecipitation and western blot analysis, it has been reported that IPO8 is physically associated with Ago2, so IPO8 may mediate Ago2 and miRNA nuclear translocation through directly binding to Ago2 [42].

Additionally, a few studies have identified a specific nuclear localization signal sequence in the nucleotide sequence of highly abundant nuclear miRNAs. Mature miRNAs that bear a nuclear localization signal (AGUGUU) at their 3′ terminus are specifically targeted to the nucleus [25]. It is plausible that the nuclear pore complex recognizes and utilizes this specific sequence to target miRNAs to the nucleus.

## Functions of Nuclear miRNAs

Nuclear miRNAs play a critical role in the overall gene regulation network due to their numerous functions. In this paragraph, we summarize these functions in three main categories: regulation of gene transcription, posttranscriptional regulation of RNA, and regulation of protein activity. In most cases, the functional activity of miRNAs is dependent on their incorporation into miRISCs. However, there are some instances, such as in the case of interaction with caspase 3, where miRNAs can act independently to elicit their functional effects.

### Regulation of gene transcription

#### Interaction with promoters

Since 2009, nuclear miRNAs have been demonstrated to have the potential to interact with gene promoters, with numerous possible binding sites residing therein [ 43‒ 45]. From then to now, numerous studies have indicated that miRNAs may regulate gene expression at the transcriptional level by binding to promoter regions, mediating either transcriptional gene activation (TGA) or transcriptional gene silencing (TGS).

##### Transcriptional gene activation

**Figure 2:**
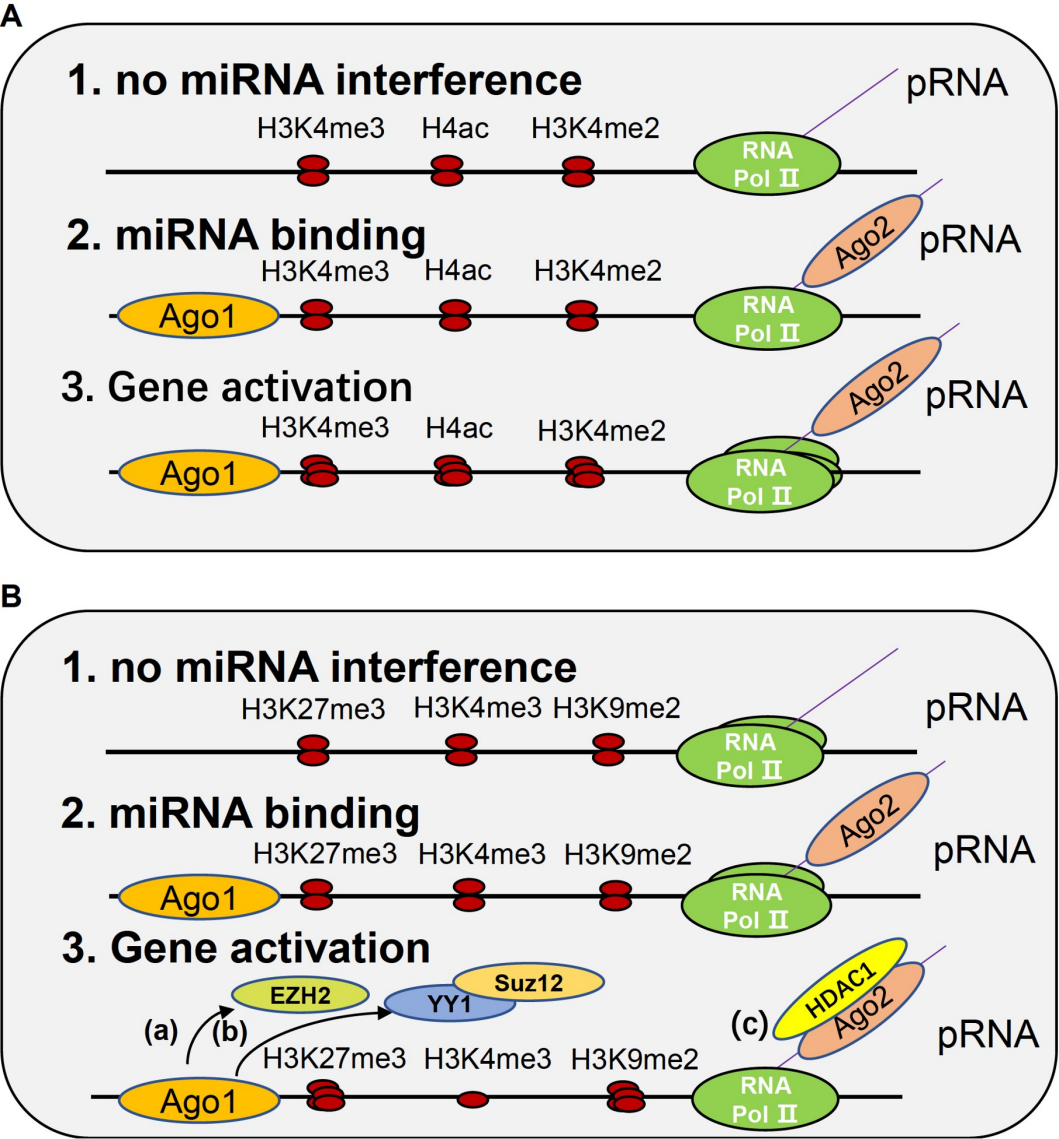
Figure 2 miRNA-mediated gene promoter regulation (A) The activating function of miRNA. First, in the steady stage, a certain amount of RNA polymerase II transcribes the promoter into pRNA. Second, Ago1 mediates miRNA binding with the promoter region, while Ago2 mediates miRNA binding with pRNA. Third, Ago2 recruits TNRC6A to form a complex, and the binding of both Ago1 and Ago2 leads to the enrichment of RNA polymerase II and active histone modifications, such as H3K4me3, H4ac (histone 4 acetylation) and H3K4me2. (B) The suppressive function of miRNA. First, in the steady stage, a certain amount of RNA polymerase II transcribes the promoter into pRNA. Second, Ago1 mediates miRNA binding with the promoter region, while Ago2 mediates miRNA binding with pRNA. Third, some modifications can be carried out. (a) Ago1 recruits EZH2 to increase H3K27me3. (b) A chromatin silent status is generated by miRNA by recruiting PcG members (YY1 and Suz12), and H3K27me3 is increased while H3K4me3 is hindered. (c) Ago2 acts as a mediator to recruit HDAC1 to increase H3K27me3 and H3K9me2 while decreasing H3K4me3. All of the above processes lead to a decrease in RNA polymerase II and gene suppression.

The precise mechanism underlying how nuclear miRNAs regulate gene expression through interactions with promoters remains unclear, but a number of hypotheses have been proposed. For TGA, two main possible mechanisms have been proposed, including miRNA influence on epigenetic modification and bidirectional transcription ( Figure 2A).

The first plausible mechanism involves miRNA-mediated epigenetic modification of gene promoters, leading to alterations in gene transcriptional activity [ 34, 46, 47]. For example, miR-744 is known to regulate the expression of mouse Cyclin B1 (Ccnb1). Studies have demonstrated that Ago1 is recruited to the Ccnb1 promoter by miR-744, and miR-744 can induce increased Pol II occupancy and H3K4me3 at the *Ccnb1* promoter, thus resulting in transcriptional activation [46] .

Additionally, miR-589 activates the expression of cyclooxygenase-2 (COX-2). In this case, miR-589 binds pRNA, which functions as a scaffold, and recruits Ago2 and GW182 (TNRC6A) to form a complex. This complex can modify histones through the assistance of WDR5, a WD40 repeat-containing protein that can act as a protein scaffold to stimulate histone methyltransferase activity. As a result, H3K4me3 at the COX-2 promoter increases, and gene transcription is activated [47].

Furthermore, a single miRNA can target several promoters to enhance the expressions of multiple genes. For instance, miR-205 targets specific sites in both *IL-24* and *IL-32* promoter RNA, increasing the expressions of both genes with assistance through inducing local active modifications, such as H3 acetylation, H4 acetylation, and H3 lysine 4 dimethylation, accompanied by the enrichment of Pol II [34].

Another possible mechanism is associated with bidirectional transcription [48]. In mammalian cells, there are numerous sense‒antisense transcript pairs. The antisense transcript of a bidirectionally transcribed gene can induce epigenetic silencing of the sense gene promoter [49]; thus, suppression of the antisense transcript can lead to gene activation. For example, the p21 antisense RNA Bx332409 directs suppressive modifications, such as H3K27me3, at the loci of the *p21* gene. A small interfering RNA (siRNA), p21-322, can significantly suppress the p21 antisense RNA Bx332409, resulting in the loss of its suppressive epigenetic modification and consequently leading to increased p21 mRNA transcript expression. Notably, Ago2 is necessary for p21-322-mediated gene activation. [48]. Although siRNA functions differently from miRNA, evidence has demonstrated that many bidirectionally transcribed genes overlap significantly with miRNAs on the 3′ UTR [50], suggesting the potential for miRNA-mediated transcriptional activation.

##### Transcriptional gene suppression

By interacting with promoters, certain nuclear miRNAs are capable of performing cotranscriptional gene suppression [ 51 ‒ 57] ( Figure 2B). One example of this is miR-552, which is capable of inhibiting human cytochrome P450 (CYP)2E1 expression both co-transcriptionally and posttranscriptionally. The seed sequence of miR-552 functions by inhibiting protein production, while the nonseed sequence is necessary for inhibition at the mRNA level. The promoter of *CYP2E1* contains an 11 nt incomplete inverted repeat and a loop hairpin with a cruciform structure, and it has been demonstrated that the cruciform structure in this promoter region plays a regulatory role in gene transcription [51]. Through binding with this cruciform structure, the nonseed sequence of miR-552 is able to inhibit SMARCE1, which is an accessory component of the mammalian SWItch/Sucrose NonFermentable (SWI/SNF) chromatin remodeling complex and is involved in transcriptional gene activation. This results in hindering the recruitment of Pol II and ultimately suppressing Pol II-dependent CYP2E1 transcription [58].

Multiple studies have found that this kind of transcriptional regulation requires the Ago1 protein. For example, miR-320 is capable of inhibiting POLR3D mRNA expression. Specifically, miR-320 directs Ago1 in a sequence-specific manner to the promoter of *POLR3D*, leading to the enrichment of histone-lysine N-methyltransferase enzyme (EZH2), a histone methyltransferase that mediates H3K27me3 [58]. Another study also reported that miR-208b can bind to EZH2 and inactivate the promoter of related genes in a similar manner [59].

Moreover, miR-223 can bind to sites within the promoter of the *NFI-A* gene and recruit Ago1, Dicer1 and PcG members (YY1 and Suz12) to this region, leading to a decrease in transcriptionally active chromatin marks acetylated H3 and H3K4me3 and an increase in inactive chromatin marks H3K27me3, as polycomb (PcGs) and trithorax (TrxGs) proteins are responsible for the trimethylation of H3K27 and H3K4, respectively [ 55, 56]. Thus, miR-223 can generate a chromatin-silent state on the *NFI-A* gene promoter during granulopoiesis, which plays an important role in directing the fate lineage determination of hematopoietic progenitors [57] .

In addition to the Ago1 protein, the Ago2 protein also seems to be associated with this miRNA-dependent transcriptional regulation. For example, miR-423-5p mimics can suppress progesterone receptor (PR) gene transcription by binding to the promoter itself and recruiting Ago2 to an antisense noncoding RNA transcribed from the PR promoter, ultimately resulting in decreased Pol II occupancy and an increased level of histone H3K9me2 at the PR promoter [52]. However, further investigations should be conducted to explain the role that the Ago protein plays and the mechanisms for miRNA-promoter transcriptional processes.

##### Three interaction models

In conclusion, three main models have been proposed for miRNAs to interact with promoters [ 60, 61]: RNA-DNA, RNA-RNA and RNA-DNA triplex. The function of the interaction can be either activation or silencing, and its function can be related to the specific target region and the epigenetic status of the promoter [62]. These interactions are also dependent on Ago proteins [63].

In the case of RNA-DNA interactions, miRNA-Ago complexes bind to the TATA box or transcription factor-binding sites of the promoter, thereby recruiting transcription factors, epigenetic modifiers and Pol II to the promoter region. For example, as mentioned above, miR-552 has been shown to directly bind with *CYP2E1* promoter DNA to suppress its transcription [58]. Similarly, miR-138, miR-92a, and miR-181d can activate promoter activities through binding to the TATA-box motifs of insulin, calcitonin, or c-myc, respectively [64].

In contrast, RNA-RNA interactions involve the binding of the miRNA-Ago complex to noncoding transcripts derived from gene promoters, wherein either sense or antisense transcripts can be targeted by miRNA-Ago complexes. This interaction then recruits histone modifiers, transcription factors and Pol II for further processing of the promoter [65].

For RNA-DNA triplex interactions, miRNA-Ago complexes directly alter the topography of chromatin, thereby changing its accessibility to transcription factors and resulting in either activation or suppression of transcription. The triplex can also suppress transcription by overlapping the target site of transcription factors on the promoter [66]. The accuracy of the triplex binding mechanism has been verified through the binding of hsa-miR-483-5p to duplex DNA *in vitro* [67].

#### Interaction with enhancers

In addition to promoter regions, recent studies have shown that miRNAs also have the ability to interact with enhancers, which are genomic elements capable of upregulating gene transcription.

Some miRNAs whose gene loci are identified within the peaks of H3K27ac modification, which is an active enhancer marker, are believed to have the potential to activate neighboring gene transcription by targeting enhancers [68]. For instance, miR-26a-1 overexpression results in an increase in transcription of neighboring *ITGA9* and *VILL* genes, and miR-339 overexpression activates the neighboring gene *GPER* by 4-fold. Researchers have further confirmed this relationship between enhancer markers and enhancer-associated miRNA function through examples of miR-3179 and miR-3180, two miRNAs located at the same intergenic region with significantly different H3K27ac enrichment levels. Transient transfection of miR-3179 into HEK293T cells results in the upregulation of neighboring genes *ABCC6* and *PKD1P1* by 3-fold and 5-fold, respectively, while miR-3180 does not show any activation [68].

**Figure 3:**
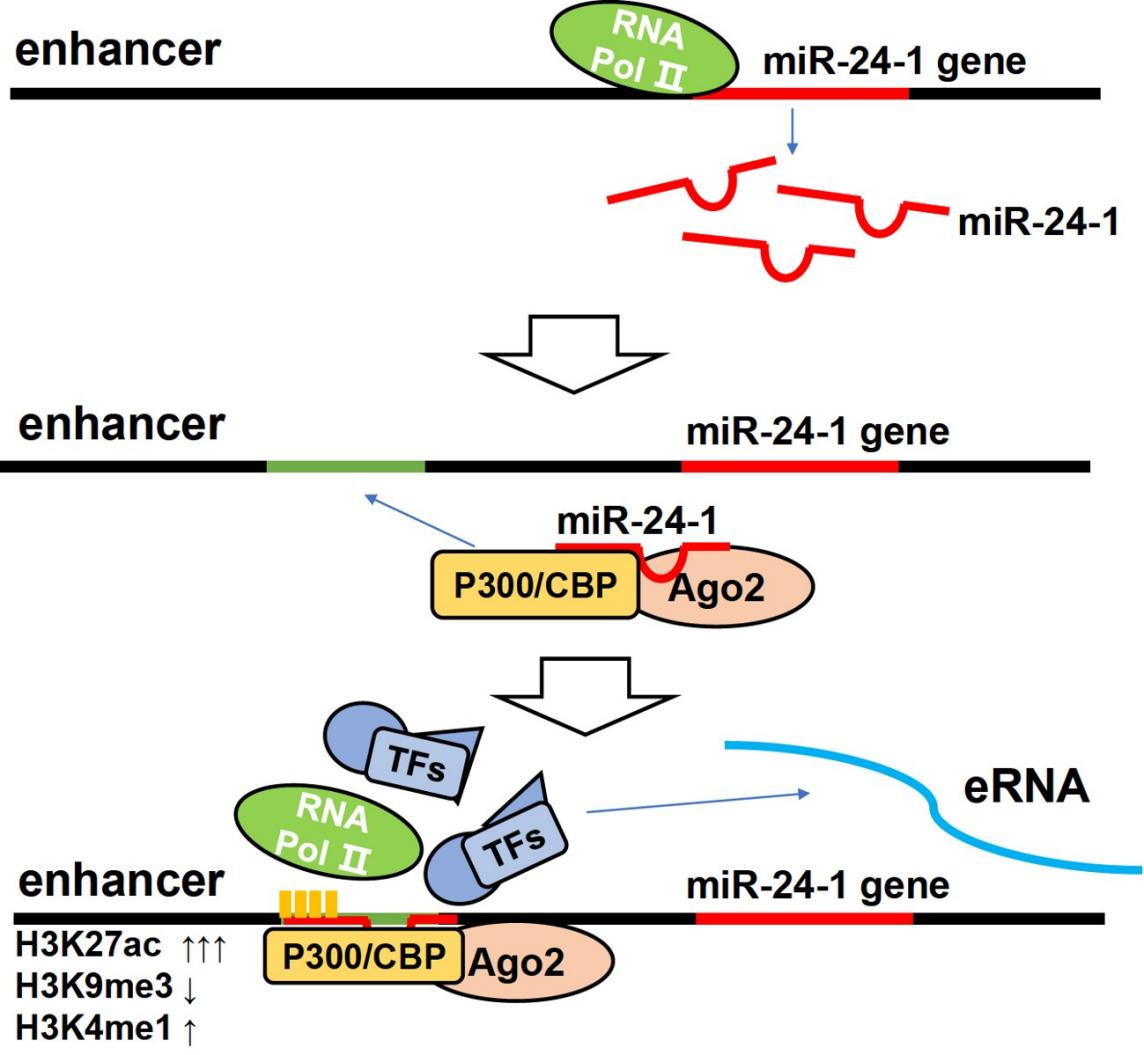
Figure 3 miR-24-1-mediated gene enhancer activation First, miR-24-1 is transcribed from its gene at enhancer loci. Second, mature miRNA forms a complex with Ago2 and p300/CBP, inducing active markers at enhancer regions, including an increase in H3K27ac and H3K9me3 and a decrease in H3K4me1, making the enhancer recognizable to RNA polymerase II. Third, TFs and other proteins bind to RNA polymerase II, and RNA polymerase l II is activated. Then, it bidirectionally transcribes the enhancer. Subsequently, eRNA binds to p300 and other proteins to activate the target gene promoter.

To explore the mechanism of miRNA as an enhancer trigger, researchers focused on the function of miR-24-1 ( Figure 3). It has been demonstrated that increased level of miR-24-1 activates the transcription of its neighboring genes *FBP1* and *FANCC*. At first, it can induce Ago2, p300/CBP and Pol II enrichment at the enhancer region. Meanwhile, the active enhancer marker H3K27ac and the poised enhancer marker H3K4me1 are increased, and the repressive marker H3K9me3 is depleted. As a result, functional enhancer RNA (eRNA) transcripts are increased, which can subsequently activate gene transcription. That is, miR-24-1 overexpression leads to direct chromatin state alteration of the enhancer, which is involved in gene activation [68].

Generally, the miRNA-enhancer target gene activation process can be summarized in three steps. First, miRNA is transcribed from its gene on enhancer loci. Second, it forms a complex with Ago2 and p300/CBP, inducing an increase in H3K27Ac and H3K4me1 levels and a decrease in H3K27me3 level at the enhancer region, making the enhancer recognizable to Pol II. Third, Pol II attracts transcription factors and proteins such as P-TEFb, PAF1 and SPT6. It is then activated through phosphorylation and bidirectionally transcribes the enhancer. Afterwards, the integrator complex cleaves the 3′UTR of eRNA transcripts, converting it to an appropriate size. Subsequently, mature eRNAs bind to p300/CBP and other proteins to induce activation markers at the target gene promoters. Pol II and other transcription factors then attach to the gene promoter and activate target gene expression [ 69, 70 ].

### Posttranscriptional regulation of RNA

#### Interaction with mRNA

After maturation, miRNAs can form miRISCs with Ago2 and other components in the cytoplasm. After being transported into the nucleus, miRISC can perform its function just as in the cytoplasm, using the guide strand to bind with certain sites called miRNA response elements (MREs) on mRNAs, finally causing target mRNA loss of function and degradation. Some of these interactions occur co-transcriptionally, and some occur posttranscriptionally. While MREs are usually on the 3′UTR of mRNAs, some are on the 5′UTR and even protein-coding sequences [ 61, 71‒ 73]. When the miRNA:MRE interaction is fully complementary, it can induce Ago2 endonuclease and cause target mRNA cleavage [74]. For animals, miRNA:MRE complementarity is usually imperfect, preventing Ago2 endonuclease activity [75].

**Figure 4:**
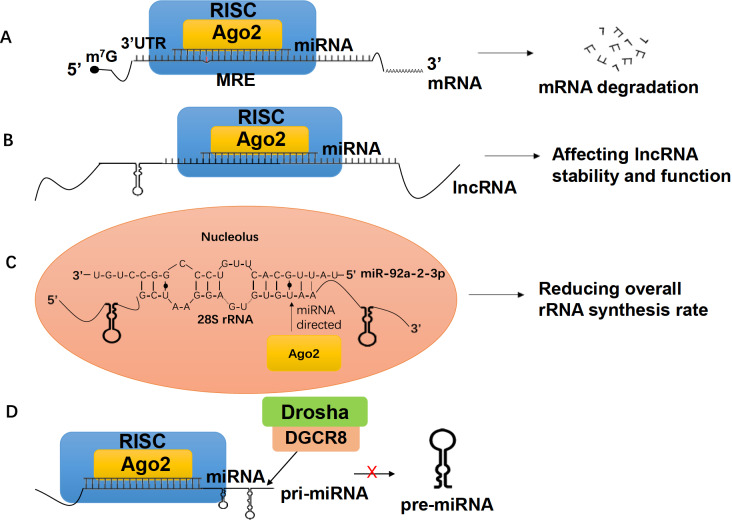
Figure 4 Posttranscriptional regulation of RNA Nuclear miRNAs participate in the posttranscriptional regulation of RNA via several mechanisms. (A) Interaction with mRNAs. Mature miRNAs can enter the nucleus, form miRISCs and bind with MREs on the 3′UTR of mRNAs. In animals, the complement system is usually imperfect. These interactions eventually lead to mRNA degradation. (B) Interaction with lncRNAs. miRISCs can target miRNA-complementary sequences on lncRNAs to affect their stability and function. (C) Interaction with rRNAs. An example of this mechanism is miR-92a-2-3p interacting with 28S rRNA. In the nucleolus, miR-92a-2-3p directs the binding of Ago2 to 28S rRNA, resulting in the downregulation of the overall rRNA synthesis rate. (D) Interaction with pri-miRNAs. Pri-miRNAs can also be the target of miRISCs. After binding with pri-miRNAs, miRISCs can prevent Drosha/DGCR8 from splicing pri-miRNA to pre-miRNA, which eventually stops miRNA maturation. This shows that certain miRNAs have higher priority and can regulate the expression of other miRNAs.

As reported, low molecular weight miRISCs can interact with mRNAs in the nucleus posttranscriptionally and induce mRNA degradation, but the mechanism is not clear yet [ 40, 76 , 77] ( Figure 4A). Additionally, it has been reported that Ago proteins and Drosha are involved in mRNA splicing [ 78, 79], supporting the existence of co-transcriptional miRISC:mRNA interactions. By directly interacting with MREs on mRNAs, miRISCs in the nucleus can regulate gene expression in many ways, contributing greatly to the whole miRNA regulatory network.

#### Interaction with lncRNA

Long noncoding RNAs (lncRNAs) are transcripts longer than 200 nucleotides with low or no protein coding potential that participate in the modulation of gene expression at both the transcriptional and posttranscriptional levels [80]. In humans and other eukaryotes, a vast proportion of the genome is transcribed in a developmentally regulated manner, resulting in the production of numerous lncRNAs.

Converging lines of evidence suggest that lncRNA expression is subject to posttranscriptional regulation by nuclear miRNAs, which bind to miRNA-responsive elements within lncRNAs, thereby impacting their stability and function ( Figure 4B). In 2011, researchers identified that miR-671, which is primarily localized in the nucleus, directs cleavage of the antisense transcript of the cerebellar degeneration-related protein 1 (CDR1) gene in an Ago2-dependent manner [81]. This was the first experimental evidence pointing towards the functional targeting of lncRNAs by miRNAs [36]. Since then, multiple nuclear lncRNAs have been identified as target genes for miRNAs. For instance, the cancer-associated nuclear lncRNA MALAT-1 has been reported as a target of miR-9 [82], while another cancer-associated nuclear lncRNA, XIST, has been reported as a target of miR-210 [83]. The GENCODE Consortium has implemented a map of putative microRNA target sites, including 10419 lncRNA genes in the current version, many of which are nuclear enriched [84]. Further experimental investigations will likely shed more light on the intricate network governing the regulation of lncRNAs by miRNAs in the cell nucleus.

#### Interaction with rRNA

In addition to the interaction with specific regions of mRNA or lncRNA, evidence suggests that nuclear miRNAs may have the potential to interact with ribosomal RNA (rRNA).

Nuclear miRNAs have been observed to be localized in the nucleolus [ 85, 86], which is composed of three distinct components: the fibrillar centers (FCs), where rDNA genes reside; the dense fibrillar component (DFC), where pre-rRNA transcripts undergo processing; and the granular component (GC), where additional pre-rRNA processing and ribosome assembly occur [87]. For instance, miR-206 has been shown to be present not only in the cytoplasm but also in the nucleolus. Furthermore, it has been demonstrated that miR-206 partially colocalizes with 28S rRNA in both the cytoplasm and granular component of the nucleolus, suggesting that miRNAs possess the capacity to bind to rRNA and potentially play a role in rRNA biogenesis [24].

Other studies have reported that Ago2, an essential component of the miRISC, can bind with rRNA. The ability of Ago2 to interact with rRNA is nearly abolished in *DICER*-knockout cells, implying that miRNAs may mediate the association between Ago2 and rRNA. Specifically, miR-92a-2-3p has been found to facilitate Ago2 cross-linking with rRNA possessing a perfect match with the seed sequence of this miRNA ( Figure 4C). Knockdown of *Ago2* in HEK293T cells led to a significant increase in overall rRNA synthesis, thereby supporting the notion that miRISC components may participate in rRNA biogenesis [37].

#### Interaction with pri-miRNA

It has long been documented that miRISC can interact with mRNA through complementary MREs, thereby mediating posttranscriptional gene silencing [ 61, 71- 73]. In a similar pathway, pri-miRNAs can also serve as targets of miRNAs ( Figure 4D).

Previous studies have shown that miR-709 can inhibit the processing of miR-15a/16-1 by binding to a 19-nucleotide miR-709 recognition element present on pri-miR-15a/16-1 [88]. Additionally, miR-122 can impede the maturation of pri-miR-21 by binding to specific nucleotides recognized by the microprocessor complex [89]. These findings suggest a hierarchical structure among miRNAs, wherein certain miRNAs hold higher priority and can regulate the expression of other miRNAs.

#### Regulation of alternative splicing

Scientific evidence demonstrates that miRNAs play a pivotal role in splicing events within gene regulatory networks. Immunoprecipitation assays have provided substantial proof of the interaction between Ago proteins and several splicing factors in the nucleus [90]. For example, crosslinking immunoprecipitation assays coupled with high-throughput sequencing (HITS-CLIP) have enabled the identification of Ago2 and miRNA binding sites within intronic sequences by various studies [ 91‒ 93]. Furthermore, splicing outcome evaluation in *DICER*-knockout cells has established that DICER-dependent small RNAs, including miRNAs, are crucial to splicing events within the cells [ 78, 90 ], further substantiating the role of miRNA in alternative splicing. However, the underlying mechanism remains poorly understood.

SF2/ASF, which is a splicing factor, can form a negative feedback loop with miR-7. Direct interaction between SF2/ASF and the primary miR-7 transcript facilitates Drosha cleavage and enhances microRNA expression, and mature miR-7 subsequently targets the 3′UTR of SF2/ASF to repress its translation [94]. This suggests that the interaction between splicing factors and specific miRNAs may be a part of the alternative splicing regulation network.

#### Detention in the nucleus to fine-tune mRNA expression

It has been proposed that miRNAs can be retained in the nucleus to fine-tune the expressions of their mRNA targets. For instance, miR-706, miR-709, and miR-690 regulate the expression of Stat1, Myc and Cebpα, respectively, all of which are transcription factors involved in myeloid differentiation [ 95‒ 97]. It is suggested that the enrichment of these miRNAs in the nucleus is linked to their decreased cytoplasmic concentration, and their retention is able to upregulate the expression of transcription factors and other proteins during granulopoiesis [97].

### Regulation of protein activity

In most situations, miRNA typically engages with Ago proteins and binds to specific RNA or DNA regions to perform either activation or suppression. It should be noted that some miRNAs can function independently of Ago proteins and directly interact with non-Ago proteins.

**Figure 5:**
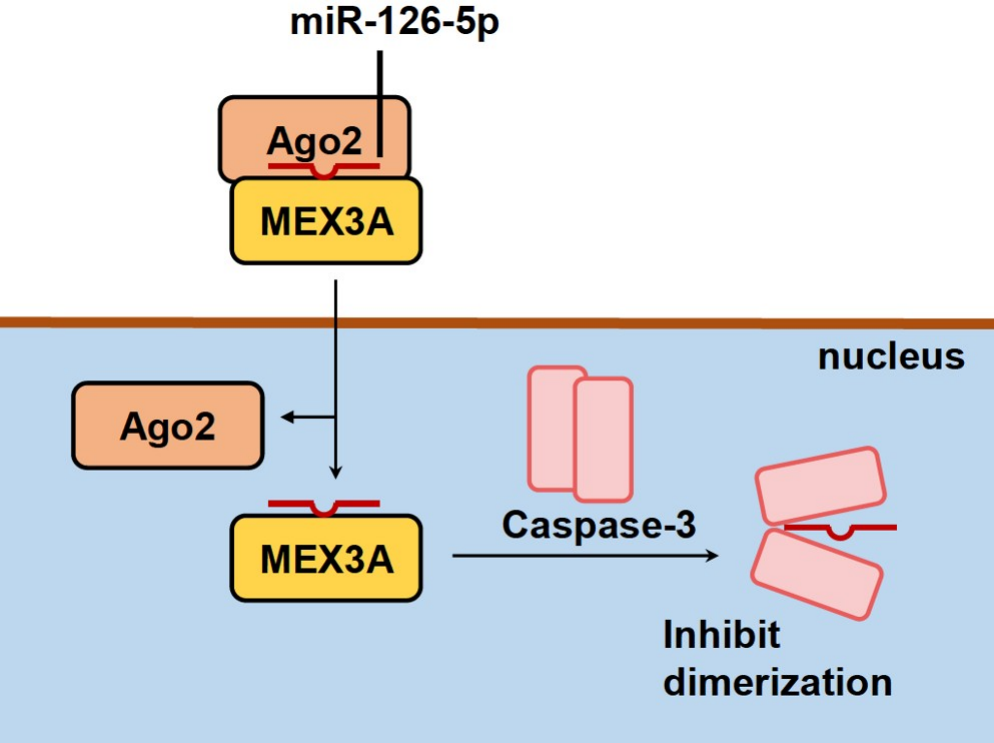
Figure 5 miR-126-5p-mediated protein activity regulation After forming a complex with Ago2 and MEX3A, miR-126-5p, with the complex, is transported into the nucleus. Ago2 then dissociates from the complex, and miR-126-5p can interact with caspase-3. It inhibits the dimerization of caspase-3 and plays a role in antiapoptosis.

For instance, miR-126-5p can serve as an aptamer to caspase 3 ( Figure 5). Upon maturation in the cytoplasm, miR-126-5p associates with Mex3a and Ago2 to form a complex that is translocated into the nucleus. In the nuclear compartment, miR-126-5p dissociates from Ago2 and binds to caspase 3 using its seed sequence. Following this interaction, the dimerization of caspase 3 is inhibited, thereby decreasing its activity in limiting apoptosis and promoting antiapoptotic function. This reveals a noncanonical mechanism by which miRNAs directly bind to target proteins and modulate protein function [98].

## Pathophysiology of miRNAs in the Nucleus

miRNAs play a critical role in the pathophysiology of multiple diseases, including neuroendocrine tumors, nonalcoholic fatty liver disease (NAFLD), and cardiovascular diseases [ 99 ‒ 104]. In the same way, it is also crucial to determine the role that nuclear miRNAs play in the pathophysiology of diseases to better prevent diseases, elucidate the mechanisms and identify novel biomarkers.

One example is nuclear miR-665, which is observed to have increased expression in heart failure. The possible pathophysiology for heart failure patients is that nuclear miR-665 targets the *PTEN* (phosphatase and tensin homolog) gene in the nucleus, thus inducing cardiac dysfunction related to TAC (transverse aortic constriction) and heart failure [105] .

Another example is nuclear miR-30b-5p. In abnormal autophagy-related diseases, the expression of miR-30b-5p is decreased in the nucleus. MiR-30b-5p can bind with coordinated lysosomal expression and regulatory elements, which are palindromic motifs associated with autophagy and lysosomes. In this way, the interaction with the transcription factor TFEB is suppressed, leading to less activation of autophagy and biogenesis of lysosomes [106].

MiR-320 is reported to be related to heart failure in type 2 diabetes mellitus patients. The expression of this kind of miRNA is increased in the failing heart of diabetes patients compared with the expression in patients without diabetes. Nuclear miR-320 can promote the transcription of the Cd36 gene and increase cardiomyocyte uptake of free fatty acids, leading to the accumulation of lipotoxic diacylglycerol and reactive oxygen species. After that, apoptosis occurs, and diabetic cardiomyopathy ensues [107].

## Databases in miRNA Research

Currently, many databases about miRNA biogenesis and functions in the nucleus have been established. Several of them are introduced as follows.

The following databases are about susceptibility loci for nuclear miRNAs. From these databases, it is possible for us to propose potential targets for nuclear miRNAs.

EnhancerAtlas 2.0 ( http://enhanceratlas.org/index.php) gives the locations of enhancers in the human genome in a genome-wide manner [108]. It also provides the target genes for a certain enhancer. This can be used to predict the enhancers near a susceptibility locus for nuclear miRNAs and list the potential target genes.

EnhancerDB ( http://lcbb.swjtu.edu.cn/EnhancerDB/) enables users to search related miRNAs, genes, TFs and SNPs through this enhancer’s name [109]. It also works when using a miRNA’s name to search related enhancer and TFs. The expression level of this miRNA will also be given.

EnhFFL ( http://lcbb.swjtu.edu.cn/EnhFFL/) can be used to search enhancer-miRNA-gene and TF-enhancer-miRNA interactions through miRNA name or enhancer name [110]. This can provide a clear picture of miRNAs in the nucleus and their corresponding enhancers.

ChIP-Atlas: Target Genes ( https://chip-atlas.org/) contains ChIP data for Ago1 and Ago2, making it possible to find potential target genes for many kinds of transcription factors [111]. As Ago2 is thought to be a core component of nuclear miRISC, these ChIP data may contain potential targets for nuclear miRNAs.

The 3Dgenome interaction viewer and database ( http://kobic.kr/3divv1/) offers data about long-range chromatin interactions, including enhancer-promoter interactions, and has the ability to visualize them [ 112, 113]. This database can help explain epigenetic variation and phenotypical polymorphisms during which nuclear miRNAs can be involved. As a result, potential targets for nuclear miRNAs can be proposed.

Other databases can help research on nuclear miRNAs by directly providing information about miRNAs.

miRbase ( http://www.mirbase.org) is a widely used database containing nearly all miRNAs that have been discovered. By searching through their name, their precursors, expression level, location on the genome and other information can be given [114]. Since most nuclear miRNAs also exist in the cytoplasm, this database contains information for both cellular and nuclear miRNAs, making it useful in providing information on nuclear miRNAs.

RNALocate ( http://rnalocate.org/) offers more than 210000 RNA-associated subcellular localizations, which can help analyze the potential target sites for miRNAs [115]. By searching miRNAs that are localized in the nucleus, information on nuclear miRNAs can be obtained.

The following databases are established mainly for research on nuclear miRNAs, and they can provide much information on the interaction between target loci and nuclear miRNAs.

miRNASNP-v3 ( http://bioinfo.life.hust.edu.cn/miRNASNP/) focuses on single-nucleotide variations, including SNPs and disease-related variations in miRNAs and their target binding sites [116]. Since SNPs in miRNAs and their binding sites have a strong impact on the effect of nuclear miRNA, data for them will be of great help for research on nuclear miRNAs.

miRdSNP ( http://mirdsnp.ccr.buffalo.edu) is a database that integrates disease-associated SNPs, miRNA target sites and diseases. With the help of this database, miRNA target sites and disease-associated SNPs on the entire 3′UTR sequence can be obtained, which can be very beneficial for studying SNPs and certain diseases [117].

microPIR2 ( http://www4a.biotec.or.th/micropir2) contains approximately 80 million predicted nuclear miRNA target sites located in promoter sequences for humans and 40 million for mice [118]. These data are helpful for research on the interaction between nuclear miRNAs and promoters. Moreover, it is convenient for comparative analysis between human and mouse target sites of nuclear miRNAs.

## Conclusions

In the conventional pathway, the function of miRNA is typically limited to the cytoplasm. With all the efforts made by researchers, it is now clear that miRNAs can serve as regulators in both the cytoplasm and nucleus through different mechanisms. In this review, we have described how nuclear miRNAs can interact with transcription factors, mRNAs, ncRNAs and proteins to implement gene regulation. Given that the importance of miRNAs in diseases such as systemic lupus erythematosus and cancer has been well established, it is clear that further exploration of these novel mechanisms will expand our understanding of the pathophysiology of these diseases and offer new avenues for treatment.

Remarkably, SNPs in nuclear miRNAs and their target genes represent a current research hotspot. By utilizing genome-wide association study (GWAS) databases and applying high-throughput sequencing technology to miRNAs, researchers can identify how a single nucleotide mutation may influence the pairing of miRNAs and their targets and the associated downstream pathway, deepening our comprehension of miRNA-mediated disease development.

However, there are still many unsolved issues in the field. For example, some transcription factors, such as CCCTC-binding factor (CTCF), octamer-binding transcription factor 4 (OCT4) and sex determining region Y-box 2 (SOX2), bind to their regulatory regions depending on epigenetic changes [ 119, 120], but for miRNAs in the nucleus, the influence of epigenetic modifications on their interaction with targets is poorly studied and may require future efforts.

It should be noted that further *in vivo* evidence is needed to support the novel mechanisms. Nonetheless, we believe that the exploration of these noncanonical miRNAs will result in novel and exciting discoveries.
